# Acupuncture might enhance word recognition scores in a patient with hearing loss: a case report and literature review

**DOI:** 10.3389/fnins.2025.1593659

**Published:** 2025-05-15

**Authors:** Ke Fang, Da Jiang, Minghui Zhao, Hantong Hu, Yang Li, Hong Gao, Jie Zhou

**Affiliations:** ^1^The Third Clinical College of Zhejiang Chinese Medical University, Hangzhou, China; ^2^Department of Acupuncture and Moxibustion, The Third Affiliated Hospital of Zhejiang Chinese Medical University, Hangzhou, China

**Keywords:** word recognition, speech audiometry, hearing loss, acupuncture, case report

## Abstract

**Background:**

Sensorineural hearing loss (SNHL) is a hearing disorder caused by damage to the inner ear, the auditory centers, or the auditory nerve in the brain. Sensorineural hearing loss not only causes an increase in the pure tone audiometry of patients, but also may lead to a decrease in word recognition scores. Although contemporary medical advancements like cochlear hearing devices and auditory aids provide some assistance to individuals with hearing loss, many patients still seek more natural and non-invasive treatment options.

**Case presentation:**

A 61-year-old male patient came to our acupuncture clinic on September 22, 2024, complaining of significant hearing deficit in his left ear, which had persisted for 1 year. Upon admission, pure tone audiometry showed severe hearing loss across all frequencies in the left ear, with a word recognition score of 64. Based on the comprehensive examination results, we diagnosed him with sensorineural hearing loss (SNHL). The patient had previously received both traditional Chinese and Western medical treatments at several hospitals, but his symptoms had not significantly improved. On October 20, 2024, after 11 sessions of acupuncture, the patient reported an improvement in his hearing, with the word recognition score in his left ear increasing from 64 to 76. On November 17, 2024, after completing 21 acupuncture sessions, the word recognition score in his left ear further advanced from 76 to 94. Ultimately, a two-month follow-up showed no recurrence of hearing loss.

**Conclusion:**

Acupuncture might serve as a promising treatment choice for improving word recognition scores in individuals suffering from hearing loss.

## 1 Introduction

Sensorineural hearing loss (SNHL) refers to hearing loss caused by the dysfunction of the inner ear cochlea or the auditory nerve. This type of hearing loss typically involves impairments in the conversion of sound signals within the inner ear and/or the transmission of these signals through the auditory nerve to the brain. In accordance with the World Health Organization’s data, over 5% of the population experiences varying degrees of hearing loss (Rzepakowska et al., 2024). Word recognition scores serve as a critical indicator of a patient’s auditory and communication function, unlike pure tone audiometry, which measures hearing thresholds, they have garnered increasing attention in the field of hearing health in recent years. With growing societal emphasis on hearing health, the importance of speech audiometry has become more prominent (Shan et al., 2024). Speech audiometry not only plays a key role in assessing children’s language development but also provides important diagnostic evidence for hearing disorders and language impairments in adults. Therefore, the effectiveness and accuracy of speech audiometry are directly related to the communication abilities and quality of life of individuals with hearing impairments. However, currently, there are still relatively few treatment methods and means available for improving speech recognition rates, and many difficulties and challenges remain. Here, we describe a patient with SNHL who successfully improved his word recognition scores through acupuncture, indicating that acupuncture may serve as an alternative treatment option. This case was documented in strict accordance with the internationally recognized CARE checklist.

## 2 Case presentation

### 2.1 History

A 61-year-old male patient came to our hospital’s acupuncture clinic on September 22, 2024, complaining of severe hearing loss in his left ear that had been going on for a year. Upon admission, the patient’s pure tone audiometry showed severe hearing loss at all frequencies in his left ear: (125-8 kHz air conduction: 55-60-65-60-60-60-80 dB; 1258 kHz bone conduction: 40-50-50-55-50 dB). After conducting a word recognition test at 30 dB above the pure tone audiometry, with 25 words tested, each worth 4 points, and calculating the accuracy rate, we determined that his word recognition score was 64. His OAE report indicated possible outer hair cell damage in the left ear, as well as abnormal function of the stapedius muscle and Eustachian tube. Otoscopic examination and tympanometry results revealed no signs of ear infection, inflammation, or abnormal middle ear function. After evaluating all the findings, we diagnosed him with sensorineural hearing loss (SNHL). There was no history of hearing loss in his family and he had received treatment from both Western and traditional Chinese medicine at several hospitals. Nevertheless, his symptoms had not improved significantly, leading him to seek acupuncture treatment at our hospital.

### 2.2 Acupuncture treatment

**Figure 1:**
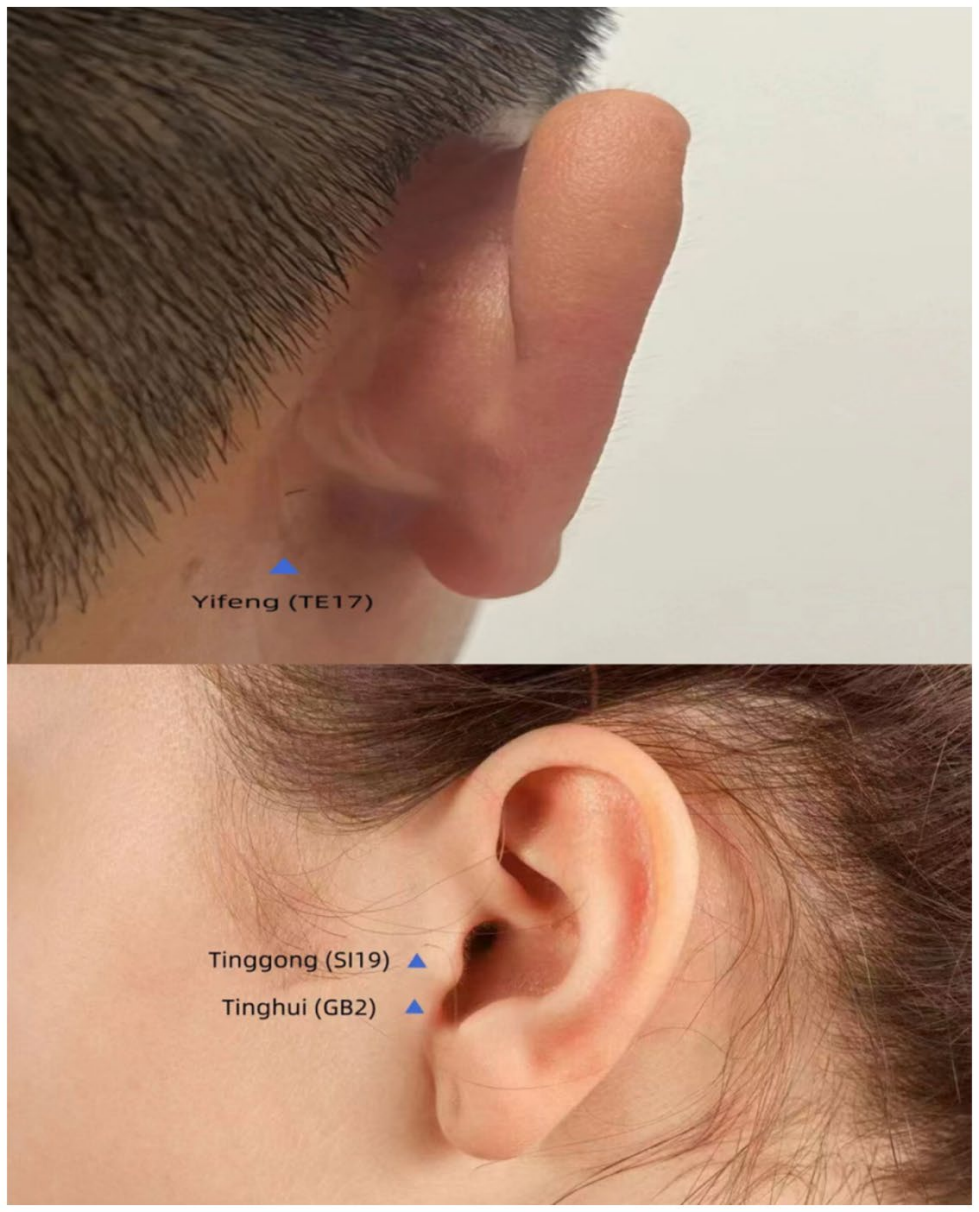
Location of acupoints (TE17) (SI19) (GB2).

**Figure 2:**
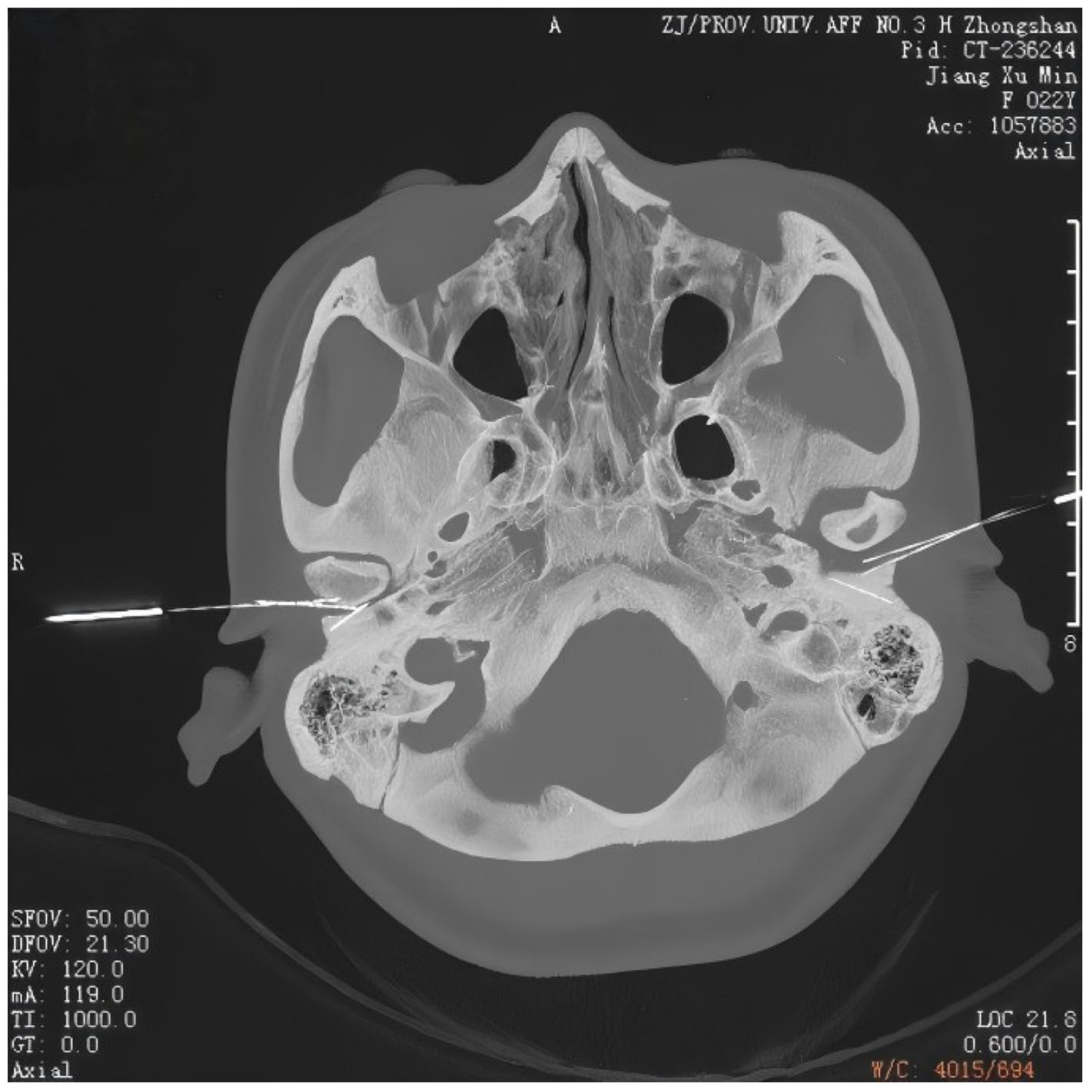
Needling depth CT image.

Regarding the acupuncture protocol, gentle acupuncture and moxibustion were performed on three traditional acupoints near the left ear (Figure 1), including Tinggong (SI19), Tinghui (GB2), and Yifeng (TE17). Tinggong, Tinghui, and Yifeng are commonly used acupoints in traditional Chinese medicine for the treatment of deafness. Many clinical reports have documented their therapeutic efficacy (Ren et al., 2024). Moreover, the acupoints of Tinggong, Tinghui, and Yifeng are closely associated with the facial nerve, and the stapedial branch of the facial nerve is closely related to hearing (Law and Corradino, 2021). Tinggong and Tinghui were inserted perpendicularly, while Yifeng was inserted obliquely, with the needle directed towards the contralateral eyeball (using 0.30 mm × 50 mm needles). The depth of needling was 25–30 mm. The needle tip was advanced to reach the temporomandibular joint (Figure 2). During the acupuncture process, a gentle and slow-paced twisting technique, characterized by an amplitude of less than 90° and a frequency of fewer than 60 twists per minute, continuously twisted for 5 to 10 s, allows the needle sensation to reach the ear and radiate deep into the ear canal. This method has a better therapeutic effect on tinnitus and is more readily accepted by patients. According to the principles of traditional Chinese medicine (TCM), the patient was diagnosed with a TCM pattern of “Liver and Kidney Yin deficiency” which was considered to be related to his hearing loss. Therefore, in addition to the aforementioned acupoints, we also used Taichong (LR3) and Taixi (KI3) to nourish the Liver and Kidney Yin. The direction of needling was also perpendicular (using 0.25 mm × 40 mm needles). It is considered a successful operation if the sensation of Qi (swelling, pain, and numbness) is conveyed into the ear canal. The operator is an experienced physician with over 10 years of acupuncture experience, ensuring the procedure’s accuracy and safety. All acupuncture points were stimulated until the Qi sensation was achieved and the needles were retained for 30 min. The patient underwent a total of 21 treatment sessions over 2 months, with treatments administered three times per week. No medication was used during the acupuncture treatment.

### 2.3 Clinical outcome

**Figure 3:**
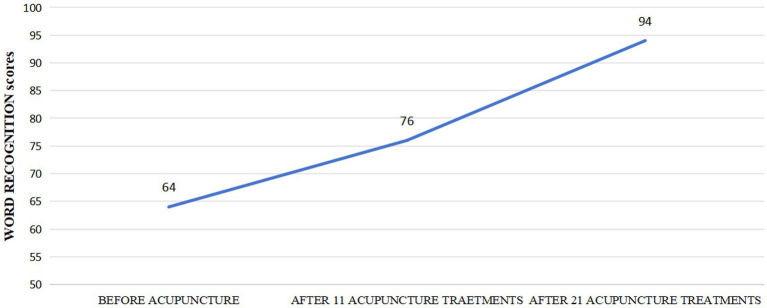
Word recognition scores from each follow-up examination.

**Figure 4:**
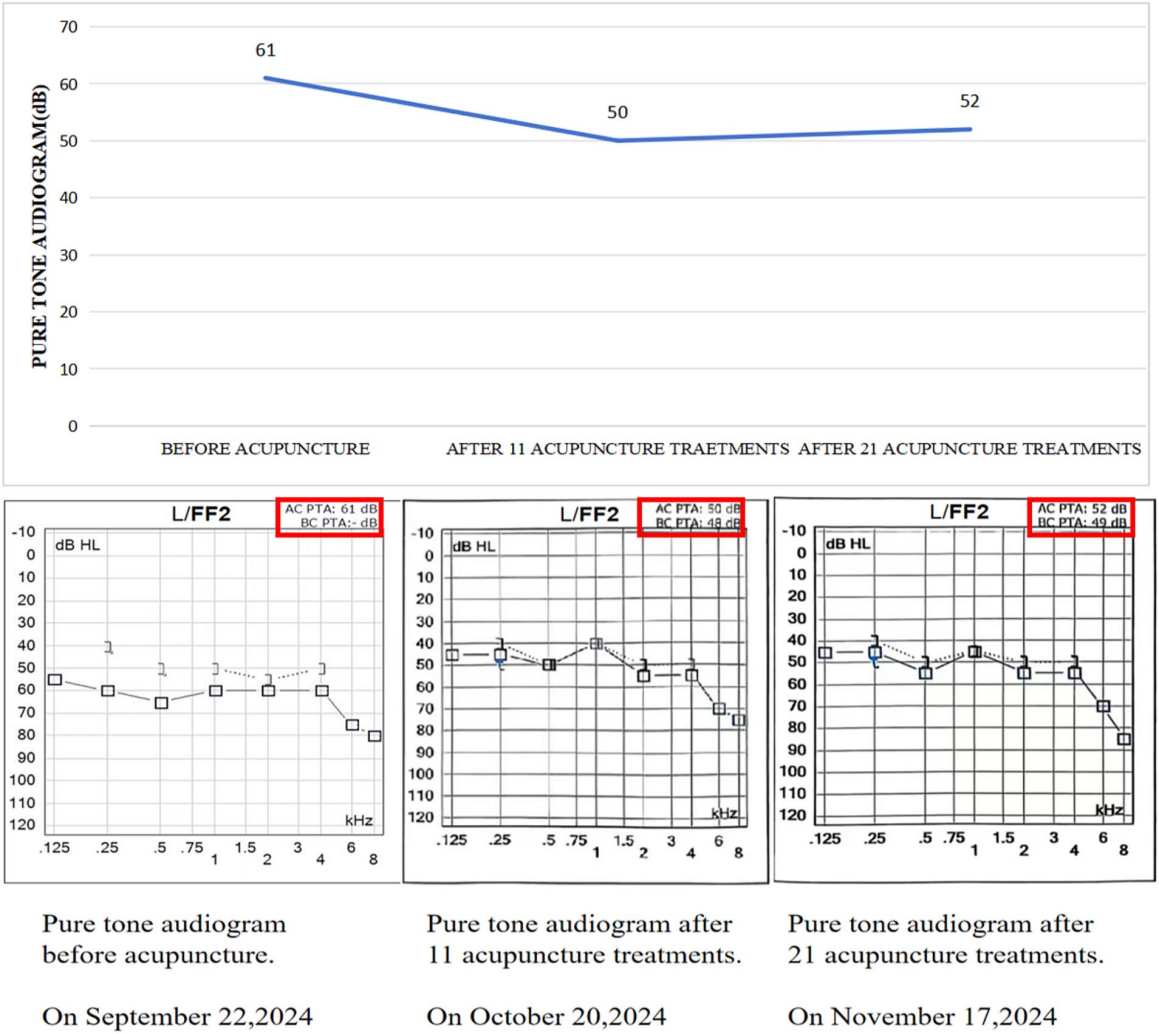
Pure tone audiogram from each follow-up examination.

After undergoing 11 acupuncture treatments on October 20, 2024, he experienced a notable improvement in his hearing ability, with his word recognition scores in the left ear rising from 64 to 76 points. On November 17, 2024, following 21 sessions of acupuncture his left ear word recognition scores rose from 76 to 94 points (Figure 3). Additionally, his follow-up pure tone audiogram displayed enhanced auditory function in the left ear (125-8 kHz air conduction: 45-45-50-70-55-55-75 dB; 125-8 kHz bone conduction: 40-50-40-50-50 dB). However, after 21 treatments, the pure tone audiogram showed no further improvement compared to the 10th session (125-8 kHz air conduction: 45-45-55-45-55-55-85 dB; 125-8 kHz bone conduction: 40-50-45-50-50 dB) (Figure 4). Ultimately, a two-month follow-up revealed no recurrence of hearing loss.

## 3 Discussion

### 3.1 The evaluation criteria and research progress in speech audiometry

Speech audiometry is an important subjective hearing test method in audiology. It assesses an individual’s ability to recognize speech by presenting standardized speech materials (such as words, short sentences, or continuous discourse) under various signal-to-noise ratio (SNR) conditions. The evaluation criteria for speech audiometry mainly include the speech recognition threshold (SRT), speech comprehension ability (such as speech recognition percentage), and speech comprehension ability in noisy environments (such as the signal-to-noise ratio, SNR, test). These criteria are established based on extensive clinical research and practical experience, aiming to provide objective and reliable assessment references for patients with various kinds of hearing loss. Specifically, the speech recognition threshold (SRT) refers to the lowest sound intensity required for an individual to correctly identify 50% of speech materials (usually disyllabic words or numbers), usually expressed in decibels (dB). It is an important indicator for assessing hearing ability, commonly used to verify the accuracy of pure-tone thresholds and to evaluate the auditory system’s sensitivity to speech. Word recognition ability is assessed through standardized speech tests, which typically include monosyllabic and disyllabic word recognition tests. The evaluation of word recognition ability in noisy environments is more complex and usually requires specific testing tools to simulate real-life noise conditions in order to assess a patient’s hearing performance in actual social situations (Dziemba et al., 2023). This case report calculates the pure-tone average hearing threshold based on the WHO 1997 classification of hearing loss. The speech presentation level is set at 30 dB SL above the pure-tone average hearing threshold of the tested ear. WRS_max_ refers to the proportion of words correctly identified by the subject out of the total number of words tested during a hearing test, under optimal hearing aid conditions. This ratio typically reaches its highest value during the hearing test and is therefore called the maximum word recognition score (WRS_max_). A WRS_max_ of ≥90% is considered the criterion for normal word recognition ability.

Recently, significant developments have emerged in the field of speech audiometry, especially in the speech comprehension abilities of patients suffering from high-frequency hearing loss. Research has shown that patients with DFNA9 carrying the COCH gene p.Pro51Ser variant have significantly lower word recognition abilities in both noisy and quiet conditions when compared to the normal-hearing comparison group. This decline in speech comprehension becomes more pronounced with increasing age. This study emphasizes the importance of not only focusing on pure-tone audiometry in clinical practice, but also valuing the assessment of speech perception, in order to provide patients with more effective hearing aids and treatment plans (Jean et al., 2025).

In terms of treatment approaches, research related to speech audiometry has demonstrated a variety of therapeutic strategies. Among these, hearing assistive devices are essential in enhancing the level of well-being for people with hearing loss. According to relevant studies, hearing aids and implantable devices (such as cochlear implants) can significantly enhance patients’ speech comprehension and social interaction abilities. For example, an analysis showed that elderly patients who received cochlear implants experienced a significant improvement in speech auditory performance (Favaretto et al., 2019). In addition, bone conduction hearing aids have also been proven to be effective for certain types of hearing loss, especially when conventional hearing aids are unable to provide sufficient assistance (Mungan Durankaya et al., 2024). Secondly, speech therapy is an important intervention for individuals with speech and language disorders. Research has shown that speech therapy can effectively improve speech comprehension and expression abilities impaired by hearing loss. For example, for children with hearing loss, speech therapy can not only enhance their language skills but also boost their social skills and self-confidence (Lieu et al., 2020).

In recent years, significant progress has been made in the field of neuroplasticity and auditory rehabilitation, particularly in the application of neuromodulation techniques and cognitive training. These advancements have brought new hope for hearing rehabilitation and enriched the assessment tools and therapeutic strategies for speech audiometry.

Neuromodulation techniques, such as transcranial magnetic stimulation (TMS) and transcranial direct current stimulation (tDCS), have shown great potential in enhancing neuroplasticity and improving auditory processing abilities in individuals with hearing loss. Transcranial magnetic stimulation (TMS) is a non-invasive neuromodulation technique that stimulates neurons in the cerebral cortex by applying a rapidly changing magnetic field to the scalp. Transcranial direct current stimulation (tDCS) is a non-invasive neuromodulation technique that modulates the excitability of neurons in the cerebral cortex by applying a weak direct current to the scalp. These techniques regulate the activity of specific brain regions involved in auditory processing, promoting the recovery of impaired auditory function and improving the efficiency of auditory signal processing, thereby significantly increasing speech recognition scores. They not only provide new treatment options for people with hearing loss but also offer a new perspective for the clinical practice of speech audiometry, making assessment and treatment more precise and personalized (Rossi et al., 2009; Ding et al., 2025).

Cognitive training has also become increasingly important in auditory rehabilitation. These programs improve the brain’s efficiency in processing auditory information through carefully designed exercises that enhance auditory attention, working memory, and the ability to distinguish speech from background noise. These training contents are closely related to speech audiometry because they directly address practical problems faced by hearing-impaired individuals, such as difficulties in understanding speech in noisy environments. Studies have shown that cognitive training can significantly improve speech comprehension, especially in challenging listening situations. This improvement is reflected not only in the objective indicators of speech audiometry but also in patients’ subjective experiences, such as enhanced social interaction skills and self-confidence (Frei and Giroud, 2025).

Combining cognitive training with traditional hearing rehabilitation methods offers more comprehensive and effective treatment options for individuals with hearing loss. Through speech audiometry assessments, doctors can more accurately understand patients’ hearing status and speech comprehension abilities, thereby developing personalized rehabilitation plans that incorporate cognitive training to enhance the brain’s processing of auditory information. This integrated approach improves patients’ speech recognition abilities and strengthens their communication skills in various environments, significantly enhancing their quality of life.

In summary, the combination of neuromodulation techniques and cognitive training has brought new hope to individuals with hearing loss. These technologies have demonstrated significant potential in basic research and have achieved practical results in clinical applications. By closely integrating with speech audiometry, these innovative technologies provide more comprehensive and effective solutions for hearing rehabilitation, helping patients better adapt to social life.

### 3.2 The clinical significance and application of speech audiometry

Speech audiometry is an important diagnostic tool that can more objectively reflect the auditory and comprehension levels of verbal sounds in patients with sensorineural hearing loss (SNHL). It can also be combined with pure-tone audiometry to evaluate the efficacy of treatment for SSNHL. The ability to receive and understand language is the core of human aural function. The word recognition rate indicates the proportion of test words or sentences that a subject can understand and correctly repeat back. This metric not only assesses the function of the peripheral auditory channel but also reveals the functional status of the auditory central nervous system. The word recognition score directly reflects the patient’s skill to recognize speech, which is crucial to quality of life and closely related to daily communication skills and social interaction abilities. Therefore, the word recognition score is an important indicator for assessing the degree of disability, social interaction ability, and the effectiveness of treatment or rehabilitation in individuals with hearing impairments.

Sensorineural hearing loss (SNHL) not only causes an increase in pure-tone thresholds but may also lead to a decrease in word recognition scores. Although both word recognition and pure-tone audiometry are subjective tests, pure-tone audiometry mainly examines the subject’s reaction to simple sounds, while word recognition evaluates how the subject responds to complex sound signals. Their response mechanisms differ. Pure-tone audiometry primarily reflects peripheral hearing function, focusing on the cochlea’s ability to detect sound at specific frequencies. However, word recognition involves more complex auditory processing, including the integration of peripheral auditory input and central nervous system function, particularly the auditory cortex.

Studies have shown that word recognition tests are superior in sensitivity to pure-tone audiometry. A decline in speech recognition ability may correspond to the damage of inner hair cells or auditory nerve fibers. It is only when the injury of auditory nerve fibers or inner hair cells exceeds 80% that the pure-tone average (PTA) begins to be affected, by which time speech recognition ability has already been severely impaired (Lobarinas et al., 2013). The pathogenesis and affected sites of sensorineural hearing loss are not yet fully understood, and lesions at any part of the auditory pathway can affect both pure-tone audiometry and speech recognition rates. For example, asynchronous firing of the auditory nerve may lead to a discrepancy between speech recognition rates and pure-tone thresholds, a phenomenon commonly seen in cases of post-cochlear and central lesions (Lan et al., 2008). This finding is consistent with our observations in clinical cases, where speech recognition scores may improve even when pure-tone thresholds do not show significant improvement. After 21 treatments, the pure-tone audiometry showed no improvement compared to the 11th session, but the speech recognition scores improved significantly.

The phenomenon of asynchrony between the periphery and the center is manifested in auditory processing research as a dysfunction between the peripheral auditory system and the central nervous system. This phenomenon is typically characterized by a decline in an individual’s ability to understand speech in complex auditory environments, despite normal peripheral hearing. For example, some studies have found that individuals with normal hearing can recognize speech well in quiet environments but exhibit significant difficulties in understanding speech in noisy backgrounds. This phenomenon is known as speech-in-noise recognition deficits (Vander Ghinst et al., 2021).

Moreover, the asynchrony between the periphery and the center can also lead to a decline in an individual’s ability to separate auditory signals in a multi-talker background. Research has shown that children with auditory processing disorders (APD) are unable to effectively distinguish sounds from different sources in complex auditory scenes, even with normal peripheral hearing. This indicates a significant difference between central processing capabilities and peripheral auditory abilities (Sidiras et al., 2020). This asynchrony has far-reaching effects on an individual’s social interactions and daily life, especially in noisy environments, where individuals struggle to understand others’ speech effectively.

The potential mechanisms underlying the asynchrony between the periphery and the center involve a variety of neurophysiological and cognitive factors. First, neurophysiological studies have shown that the normal functioning of the peripheral auditory system does not necessarily guarantee the effective operation of the central nervous system. In some individuals with neurocognitive impairments, such as those who are HIV positive, significant central auditory processing deficits exist despite normal peripheral hearing. This may be related to damage to the central nervous system (Zhan et al., 2022).

### 3.3 The mechanisms of acupuncture in enhancing word recognition scores

To date, therapeutic choices for improving word recognition ability are still restricted. This case report, however, presents an innovative approach. Following 21 acupuncture sessions, the patient’s clinical manifestations were markedly alleviated and remained stable throughout the follow-up period. Consequently, our findings indicate that acupuncture could be a promising treatment modality for enhancing word recognition scores in individuals with hearing loss.

In this case, several factors may contribute to the effectiveness of acupuncture. The fundamental principle of acupuncture mainly involves stimulating particular acupoints to modulate the circulation of qi and blood within the body, thereby achieving therapeutic effects. The mechanisms of acupuncture involve multiple physiological systems, especially the central nervous system (CNS) and the autonomic nervous system (ANS). Research indicates that the improvement of speech function by acupuncture may be linked to its modulatory effects on the central nervous system. By stimulating the CNS, acupuncture can modulate neural pathways associated with auditory and language processing, thereby enhancing speech recognition ability. For example, studies have shown that acupuncture can regulate brain neural networks to enhance the processing of auditory information, thus improving the accuracy of speech comprehension (Yang et al., 2023; Li Y. W. et al., 2022). Acupuncture, through techniques such as lifting and rotating the needles, conveys a sense of Qi to the ear canal. This enhances the neural plasticity of the auditory center, including primary auditory cortex, secondary auditory cortex, and the brain stem (Chang et al., 2022), and increasing cochlear blood flow (Cai et al., 2019). It also regulates immune function, bidirectionally modulates the immune system to sustain immune homeostasis, which mechanisms involved entail enhancing the function of NK and CD8^+^ T cell function (Ding et al., 2014; Liu et al., 2023). Studies have shown that acupuncture can enhance the auditory cortex’s response to sound stimuli. It also promotes the sensitivity and recognition ability of the auditory cortex for speech signals, thereby enhancing word recognition scores (Dewey and Shera, 2023; Köles et al., 2019). Additionally, acupuncture may also affect the release of neurotransmitters, regulate the excitability of the auditory center, and thus improve the efficiency and accuracy of auditory information processing (Oh et al., 2024; Intskirveli et al., 2024). In addition, acupuncture can also increase the expression of brain-derived neurotrophic factor (BDNF), which is crucial for neural plasticity, memory, and learning (Dilinuer et al., 2024). Additionally, research employing magnetoencephalography (MEG) have found that auditory cortex reorganization occurs within days after the occurrence of sudden sensorineural hearing loss (SSNHL) (Po-Hung Li et al., 2003; Morita et al., 2007; Suzuki et al., 2002), accompanied by decreased glucose metabolism or reduced primary auditory cortex blood flow (Okuda et al., 2013; Ueyama et al., 2015; Lanting et al., 2009). Micarelli et al. (2017) used PET to study the metabolic process of ^18^F-fluorodeoxyglucose (FDG) uptake in the brains of patients with unilateral sudden hearing loss. They found that the maximum recognition rate of disyllabic words in these patients was positively correlated with the relative shifts in ^18^F-FDG uptake in the relevant brain cortical areas. This further demonstrates the association between improved speech recognition and cortical reorganization in these areas. Acupuncture can promote auditory cortex reorganization in the brain, enhance neural conduction, and thereby improve speech function.

Relevant studies have shown that acupuncture can not only improve speech abilities but also enhance cognitive functions, especially among patients with mild cognitive impairment. Researchers have found that acupuncture can stimulate areas of the brain associated with language processing, such as the left frontal and temporal lobes, which play key roles in speech comprehension and expression (Zhou and Jin, 2008; Li B. et al., 2022). The role of acupuncture in improving speech comprehension is mainly reflected in its ability to enhance auditory attention. Studies have shown that acupuncture could modulate brain neural activity and enhance the processing of auditory information. For example, in a study on patients with hearing loss, acupuncture was found to significantly improve auditory thresholds. By activating the brain’s attention network, acupuncture helps individuals focus better on auditory information, thereby enhancing speech comprehension. This mechanism can be verified through techniques like functional magnetic resonance imaging (fMRI), which shows significantly increased activity in auditory-related brain regions after acupuncture (Zhang K. et al., 2024; Li et al., 2021). The impact of acupuncture on brain regions associated with language processing is another important mechanism through which it improves speech recognition ability. Research has demonstrated that acupuncture can enhance the activity in brain regions related to language processing by stimulating specific acupoints. For example, acupuncture targeting temporal lobes and the frontal of the brain, which are closely related to language comprehension and expression, has been found to significantly improve language abilities. In a study on patients with Alzheimer’s disease, significant improvements in language skills were observed after acupuncture treatment (Zhang S. Q. et al., 2024).

### 3.4 Future directions and clinical potential of acupuncture research

When discussing the neurobiological mechanisms of acupuncture, we must acknowledge that research on its mechanisms of action is still in the exploratory phase. Existing research has shown that acupuncture could influence brain language and auditory processing areas by regulating neural conduction and promoting neuroplasticity. The elucidation of this mechanism provides a biological foundation for using acupuncture in neurorehabilitation. However, there are certain differences among studies, such as the specific acupuncture points, stimulation intensity, and treatment frequency, all of which may affect the evaluation of therapeutic effects. Therefore, future research needs to conduct larger-scale randomized controlled trials based on standardized treatment protocols to systematically verify the specific effects of acupuncture in improving speech and auditory functions.

Moreover, the potential of acupuncture as an adjunctive therapy also deserves attention. With the development of modern medicine, single therapeutic approaches often fail to meet the needs of complex diseases. Combining acupuncture with other therapeutic modalities, such as speech therapy and auditory training, may produce synergistic effects and enhance rehabilitation outcomes. By integrating different treatment methods, we can more comprehensively address language and auditory disorders and thereby improve patients’ quality of life.

Overall, existing clinical trials have provided preliminary evidence supporting the application of acupuncture in improving speech recognition scores, but additional high-quality randomized controlled trials are still required to further confirm its effectiveness and mechanisms. Although research on acupuncture’s role in improving speech recognition scores is still in its early stages, it lays the foundation for further research and clinical application of acupuncture. We look forward to more future high-quality research that can fill the gaps in current knowledge and clarify the function of acupuncture in addressing language and auditory disorders. At the same time, we hope that acupuncture can be used as an effective adjunctive therapy, combined with modern medicine, to provide more comprehensive treatment options for patients. In summary, acupuncture shows broad prospects in the field of language and auditory disorders and is worthy of continued in-depth exploration and research.

By analyzing the existing literature, we can observe the many research advancements and potential applications in this field. However, despite the achievements made, there are still many challenges that need to be overcome. Overall, the progress in speech audiometry research and the diversification of treatment methods have shown great promise in improving individuals’ speech comprehension and communication abilities. Future research should persist in exploring the relationship between speech audiometry and hearing health, and explore more effective assessment and treatment strategies to better serve those in need.

## 4 Conclusion

Acupuncture appears to improve the word recognition ability of patients with hearing loss, but a single, uncontrolled case study is not sufficient to provide a definitive conclusion. Additional research is necessary to explore the effectiveness of acupuncture in treating hearing loss and its underlying mechanisms.

## Patient perspective

After undergoing 11 acupuncture sessions, the patient gave feedback on the treatment he received, saying, “After the acupuncture treatment, my symptom of not being able to hear clearly when chatting with others has significantly improved.” After 21 sessions of acupuncture treatment, he said: “I believe acupuncture has improved my quality of life.” In the 2 months following the end of the acupuncture treatment, there has been almost no recurrence.”

## Data Availability

The datasets presented in this article are not readily available because of ethical and privacy restrictions. Requests to access the datasets should be directed to the corresponding author.
