# Supplementary material for: Acupuncture might enhance word recognition scores in a patient with hearing loss: a case report and literature review
Source: Front Neurosci. 2025 May 15;19:1593659. doi: 10.3389/fnins.2025.1593659 (PMC12119612; doi:10.3389/fnins.2025.1593659)
Supplement: Supplementary file 1 [file Data_Sheet_1.pdf]

CARE case report guidelines

CARE Checklist of information to include when writing a case report

(cc) BY-NC-ND

Topic	Item	Checklist item description	Reported on Line
Title	1	The diagnosis or intervention of primary focus followed by the words "case report"	Lines 1-2 / Page 1
Key Words	2	2 to 5 key words that identify diagnoses or interventions in this case report, including "case report"	Line 13 / Page 1
Abstract (no references)	3a	Introduction: What is unique about this case and what does it add to the scientific literature?	Lines 26-30 / Page 1
	3b	Main symptoms and/or important clinical findings	Lines 22-24 / Page 1
	3c	The main diagnoses, therapeutic interventions, and outcomes	Lines 24-30 / Page 1
	3d	Conclusion—What is the main "take-away" lesson(s) from this case?	Lines 31-32 / Page 1
Introduction	4	One or two paragraphs summarizing why this case is unique (**may include references**)	Lines 34-48 / Page 2
Patient Information	5a	De-identified patient specific information.	Lines 51-52 / Page 2
	5b	Primary concerns and symptoms of the patient.	Lines 51-56 / Page 2
	5c	Medical, family, and psycho-social history including relevant genetic information	Line 59 / Page 2
	5d	Relevant past interventions with outcomes	Lines 59-60 / Page 2
Clinical Findings	6	Describe significant physical examination (PE) and important clinical findings.	Lines 51-58 / Page 2
Timeline	7	Historical and current information from this episode of care organized as a timeline	Lines 77-85 / Page 3
Diagnostic Assessment	8a	Diagnostic testing (such as PE, laboratory testing, imaging, surveys).	Lines 52-58 / Page 2
	8b	Diagnostic challenges (such as access to testing, financial, or cultural)	Not Applicable
	8c	Diagnosis (including other diagnoses considered)	Lines 58-59 / Page 2
	8d	Prognosis (such as staging in oncology) where applicable	Lines 84-85 / Page 3
Therapeutic Intervention	9a	Types of therapeutic intervention (such as pharmacologic, surgical, preventive, self-care)	Lines 62-75 / Page 2
	9b	Administration of therapeutic intervention (such as dosage, strength, duration)	Lines 65-75 / Page 2
	9c	Changes in therapeutic intervention (with rationale)	Not Applicable
Follow-up and Outcomes	10a	Clinician and patient-assessed outcomes (if available)	Lines 77-85 / Page 3
	10b	Important follow-up diagnostic and other test results	Lines 78-85 / Page 3
	10c	Intervention adherence and tolerability (How was this assessed?)	Lines 77-84 / Page 3
	10d	Adverse and unanticipated events	Not Applicable
Discussion	11a	A scientific discussion of the strengths AND limitations associated with this case report	Lines 212-244 / Page 6
	11b	Discussion of the relevant medical literature **with references**.	Lines 105-124 / Page 3,4
	11c	The scientific rationale for any conclusions (including assessment of possible causes)	Lines 158-211 / Page 4,5
	11d	The primary "take-away" lessons of this case report (without references) in a one paragraph conclusion	Lines 245-249 / Page 6
Patient Perspective	12	The patient should share their perspective in one to two paragraphs on the treatment(s) they received	Lines 250-255 / Page 6,7
Informed Consent	13	Did the patient give informed consent? Please provide if requested	Yes ☑ No ☐
